# Identification of metabolic biomarkers and pathways associated with *Ichthyophonus hoferi* infection in White stumpnose (*Rhabdosargus globiceps*)

**DOI:** 10.3389/fphys.2026.1826309

**Published:** 2026-06-30

**Authors:** Innocent Siyanda Ndlovu, Kevin W. Christison, Dalen Vosloo, Andre Vosloo

**Affiliations:** 1 School of Life Sciences, University of KwaZulu-Natal, Westville, Durban, South Africa; 2 Non-Communicable Diseases Research Unit, South African Medical Research Council, Cape Town, South Africa; 3 Department of Forestry, Fisheries and the Environment, Private Bag X2, Vlaeberg, Cape Town, South Africa

**Keywords:** biomarkers, fish parasite infection, *Ichthyophonus hoferi*, metabolomics, White stumpnose

## Abstract

**Introduction:**

*Ichthyophonus hoferi* is a cosmopolitan parasite infecting a wide range of fish species across marine, estuarine, brackish, and freshwater environments. Currently, diagnosis relies on destructive sampling methods, posing challenges for disease surveillance in aquaculture and wild populations. This study aimed to identify pre-clinical metabolic markers of *I. hoferi* infection in White stumpnose fish (*Rhabdosargus globiceps*) using non-lethal blood-based diagnostics.

**Methods:**

An untargeted metabolomics approach employing comprehensive two-dimensional gas chromatography time-of-flight mass spectrometry (GCxGC-TOFMS) was conducted on dried blood spot (DBS) samples. Multivariate (PCA, PLS-DA) and univariate analyses were used to evaluate metabolic differences between infected and control groups.

**Result:**

A total of 655 metabolites were detected, with 98 showing significant variation between groups. Infected fish exhibited elevated levels of xenobiotics and altered carbohydrate metabolism, with key downregulated metabolites including D-mannose, arabinofuranose, and pento-3-ulose. Several fatty acids, such as oleic acid and pentadecanoic acid, were also significantly altered. Pathway analysis revealed that propanoate metabolism, galactose metabolism, inositol phosphate metabolism, and the TCA cycle were significantly impacted by *I. hoferi* infection, suggesting disruption in energy production and anti-inflammatory pathways. Among the top discriminatory metabolites, D-mannose was notably downregulated and identified as a potential biomarker for *I. hoferi* infection.

**Discussion:**

This study demonstrates that metabolomics, in combination with DBS sample collection, offers a promising non-destructive approach for detecting parasitic infections in fish. The findings contribute to the development of diagnostic tools for early detection and monitoring of *I. hoferi* infection, with implications for fish health management and conservation.

## Introduction

Fish and fisheries play a pivotal role in global food security, economic development, and employment, supporting both skilled and semi-skilled workers (Akpaniteaku et al., 2005; Béné et al., 2007). However, disease outbreaks pose a significant threat to the aquaculture industry, with fungal and bacterial infections being among the most critical challenges (Mishra et al., 2017; Alfred et al., 2020). Fungal infections are particularly common in many fish species and can be fatal if not diagnosed and treated promptly (El-khatib et al., 2014).

Globally, fungal and fungal-like parasites are responsible for declines in fish populations. For instance, *Saprolegnia parasitica* has caused annual mortality rates of up to 50% in Coho salmon in Japan (El-khatib et al., 2014), while *Ichthyophonus hoferi* is known to induce proliferative and systemic disease in both freshwater and marine fish (Storesund et al., 2022). *Ichthyophonus hoferi* is a systemic, cosmopolitan, and obligatory mesomycetozoan parasite that has been reported in over 100 species of freshwater, estuarine, brackish, and marine fish (Hershberger et al., 2002; Hontoria et al., 2013). The disease it causes, ichthyophoniasis, has been a subject of taxonomic debate. Initially classified as a fungus (Rahimian and Thulin, 1996), phylogenetic analyses have since placed it within the Mesomycetozoea clade, alongside genera such as *Psorospermium, Rhinosporidium*, and *Dermocystidium*, organisms that lie at the evolutionary boundary between animals and fungi (Hershberger et al., 2002).

Ichthyophoniasis was experimentally induced by Hofer in 1983 in cultured Brown trout (*Salmo trutta*) and Brook trout (*Salvelinus fontinalis*) (Sitja-Bobadilla and Alvarez-Pellitero, 1990). The disease is of economic concern due to its widespread occurrence and its role in the mortality of commercially important fish species such as Atlantic herring in the western North Atlantic, as well as salmonids and farmed trout in the United States, Europe, and Japan (Kocan et al., 2009). The distribution of *I. hoferi* has also been documented across various fish species in the North Atlantic (Paperna, 1986). In Southern Africa, infections have been reported in grey mullet (*Mugil cephalus* and *Chelon* sp. (formerly *Liza* sp.) from the Kowie Lagoon (Paperna, 1986). In Cape Town, South Africa, *I. hoferi* was detected at the Two Oceans Aquarium in white stumpnose (*Rhabdosargus globiceps*) (Wurdeman, 2019).

*Ichthyophonus hoferi* infections are typically acquired through exposure to spores from infected fish (Hershberger et al., 2008; Kocan, 2019), or in freshwater aquaculture systems via feed derived from infected marine fish (Radosavljevic et al., 2024). Environmental factors such as salinity and water temperature significantly influence infection rates in waterborne transmission (Jafarizadeh et al., 2014). Failure to sterilize or disinfect contaminated feed can result in the transmission *of I. hoferi* to farmed fish (Paperna, 1986; Jones and Dawe, 2002; Hontoria et al., 2013). Additionally, the parasite may spread through predation or scavenging of infected hosts (Jones and Dawe, 2002). While schizonts are infectious when injected into fish, they appear non-infectious when administered orally, suggesting a more complex transmission route, potentially involving a paratenic invertebrate host (Kocan, 2019; Hershberger et al., 2002).

Pathologically, *I. hoferi* infection results in granulomatous lesions and systemic organ damage, often leading to mortality (Wurdeman, 2019; Radosavljevic et al., 2024). The heart is particularly affected, with the parasite causing severe cardiac muscle degeneration and ultimately heart failure (Jafarizadeh et al., 2014; Kocan et al., 2006). Several diagnostic techniques are employed, including external/internal examination, tissue squash preparation (Yanong, 2003), *in-vitro* culture of infected tissue, histopathology and PCR (Huntsberger et al., 2017; Nylund, 2022; Whipps and Kent, 2006; White et al., 2013). However, all currently available methods are destructive, necessitating the euthanasia of affected specimens.

This highlights the need for sensitive, non-lethal diagnostic alternatives. Metabolomics has emerged as a promising approach for monitoring disease and physiological stress in aquatic organisms (Roques et al., 2020). Metabolomics involves the comprehensive analysis of small-molecule metabolites in biological samples using advanced analytical techniques coupled with multivariate statistical analysis (Saoi and Britz-McKibbin, 2021; Tyagi et al., 2021; Chen et al., 2022). It has been successfully applied in human medicine to identify biomarkers for various diseases, including liver and kidney disorders (Ranjbar et al., 2015; Zhang et al., 2016). Metabolomics is particularly valuable for early detection, diagnosis, prognosis, and therapeutic discovery (Hernandez-Baixauli et al., 2020; Pereira et al., 2022; Galal et al., 2022).

This study aimed to identify preclinical metabolic markers associated with *I. hoferi* infection in white stumpnose (*Rhabdosargus globiceps*), a key species in South African recreational lagoon fisheries. Specifically, we sought to: (1) identify metabolic pathway alterations linked to infection, (2) explore the potential of metabolomics in combination with Dried Blood Spot (DBS) cards as a non-lethal diagnostic tool, and (3) characterize the metabolic response of infected fish using an untargeted metabolomics approach via GC×GC-TOF-MS analysis of incidental DBS samples.

## Materials and methods

### Study design

Incidental dried blood spot (DBS) samples were collected from White stumpnose (*Rhabdosargus globiceps*) at the Two Oceans Aquarium (Cape Town, South Africa) during an outbreak of *Ichthyophonus hoferi*. Sample collection and infection confirmation were conducted by the resident veterinarian. A total of 28 samples were categorized into two groups: (i) *I. hoferi*-positive (n = 21), and (ii) uninfected controls (n = 7). Fish were measured for total weight (2083.77 ± 1010.91 g) and length (47.33 ± 8.61 cm). Metabolomic profiling was performed using an untargeted two-dimensional Gas Chromatography Time-of-Flight Mass Spectrometry (GC×GC-TOFMS) approach.

### Diagnosis of *Ichthyophonus hoferi*

Four diagnostic techniques were employed: (i) external examination of clinical symptoms (ii) tissue squash; (iii) histology (Franco-Sierra and Alvarez-Pellitero, 1999); and (iv) PCR (Whipps and Kent, 2006; Hamazaki et al., 2013); Since some of these methods lack specificity, a positive diagnosis required positive results in at least three out of four methods.

### DBS collection and storage

Blood was drawn from the caudal vein using a 1 mL syringe and spotted directly onto Whatman 903 filter paper. Each card absorbed approximately 20-30 μL of blood per spot. Cards were labeled, air-dried at 18-20 °C for 4 hours, and stored with desiccant in gas-impermeable containers at -20 °C until analysis (Elbin et al., 2011; Benyshek, 2010).

### Sample shipping

DBS samples were stored in Cape Town at -20 °C. They were transported to the University of KwaZulu-Natal and subsequently to the National Metabolomics Platform (North-West University) on dry ice (-78 °C). Ethics approval was granted by the UKZN Animal Research Ethics Committee (AREC/030/016).

### Metabolite extraction

Samples were homogenized in 45% ethanol using a MM 400 mixer mill (Retsch GmbH) at 30 Hz for 5 minutes. Following solvent evaporation, 50 μL of 3-phenyl butyric acid (internal standard) was added. Samples were extracted in 300 μL acetonitrile on ice, centrifuged (25,000×g, 4 °C, 10 min), and the supernatant dried under nitrogen. Derivatization involved 25 μL methoxyamine hydrochloride in pyridine (15 mg/mL) at 50 °C for 90 min, followed by 84 μL BSTFA with 1% TMCS at 60 °C for 60 min (Olivier, 2012; Du Preez and Loots, 2013).

### GC×GC-TOFMS analysis

Chromatographic analysis was performed using a Pegasus GC×GC-TOFMS (LECO Corporation) with an Agilent 7890A GC. Injection volume was 1 μL at a 1:50 split. Helium carrier gas flowed at 1 mL/min. The primary column (Restek Rxi-5Sil MS) oven temperature started at 70 °C for 2 min, ramped 4 °C/min to 300 °C, and held for 2 min. A secondary Rxi-17 column facilitated 2D separation. Mass spectra were acquired at 70 eV, 1600 V detector voltage, and 200 spectra/sec (Du Preez and Loots, 2013).

### Peak identification and data processing

ChromaTOF software (v4.50, LECO) was used for peak deconvolution (S/N = 100, min 3 apexing peaks). Peaks were identified via mass spectral matching NIST mainlib and replib databases. Data clean-up included QC-based filtering for features with RSD >50%, zero-replacement with half-minimum values, log transformation, and autoscaling (Alonso et al., 2015).

### Multivariate and pathway analysis

Preprocessing steps (alignment, baseline correction) were performed in MATLAB using custom scripts. Retention indices were determined from n-alkanes and matched to spectra from NIST, the Umeå Plant Science Center, and Max Planck Golm databases (Jiye et al., 2005). Significant metabolites (n=55) were classified by chemical class using KEGG and PubChem. A metabolic map and enrichment analysis were created via MetaboAnalyst 2.0 and KEGG Mapper.

### Statistical analysis

MetaboAnalyst and SPSS v25 were used for statistical evaluations. Fold change and Mann-Whitney U tests identified discriminant metabolites (p < 0.05, fold change ≥ 2, Cohen’s d ≥ 0.8, VIP ≥ 1). PCA and PLS-DA were conducted for dimensionality reduction and biomarker identification. Data were log-transformed (shifted natural log) and autoscaled prior to analysis.

## Results

### Untargeted metabolomics profiling

An untargeted metabolomic analysis was conducted using GC×GC-ToF-MS on a total of 37 samples, comprising 9 quality control (QC) samples and 28 experimental samples. The analysis yielded 655 distinct metabolic features. Tight clustering of the QC samples confirmed the analytical stability and reproducibility of the experimental conditions. The detected metabolites encompassed a wide range of compound classes, including carbohydrates, fatty acids, xenobiotics, and other analytes in varying abundances. Both Principal Component Analysis (PCA) and Partial Least Squares Discriminant Analysis (PLS-DA) were applied to explore overall patterns of metabolic variation, with PLS-DA offering additional insights into the variables contributing to group separation.

### Metabolite variation analysis

**Figure 1:**
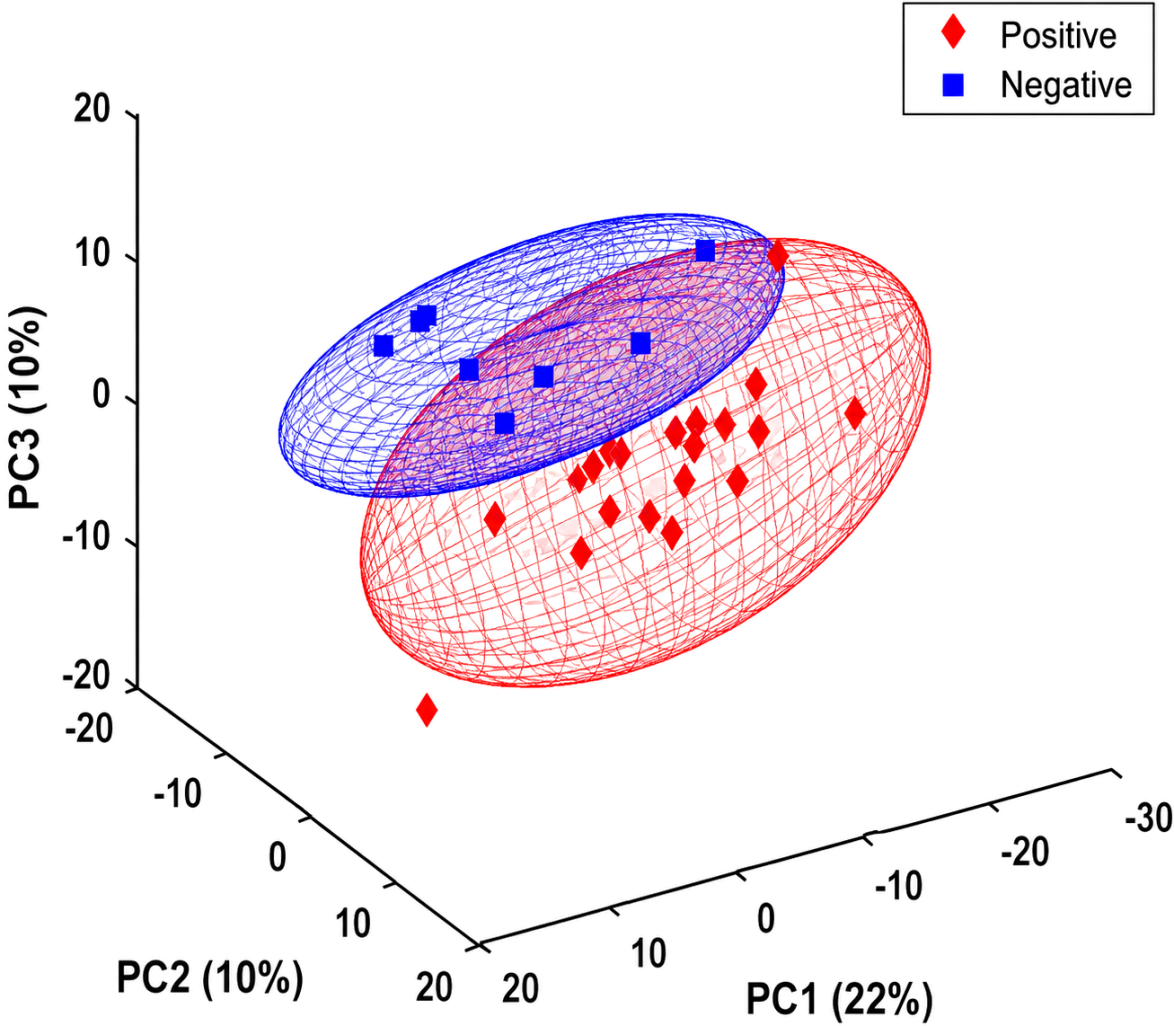
PCA score plot differentiating the control from the *I. hoferi-*positive from metabolites of White stumpnose (*Rhabdosargus globiceps)* incidental samples with ellipsoids representing 90% Confidence Intervals.

**Figure 2:**
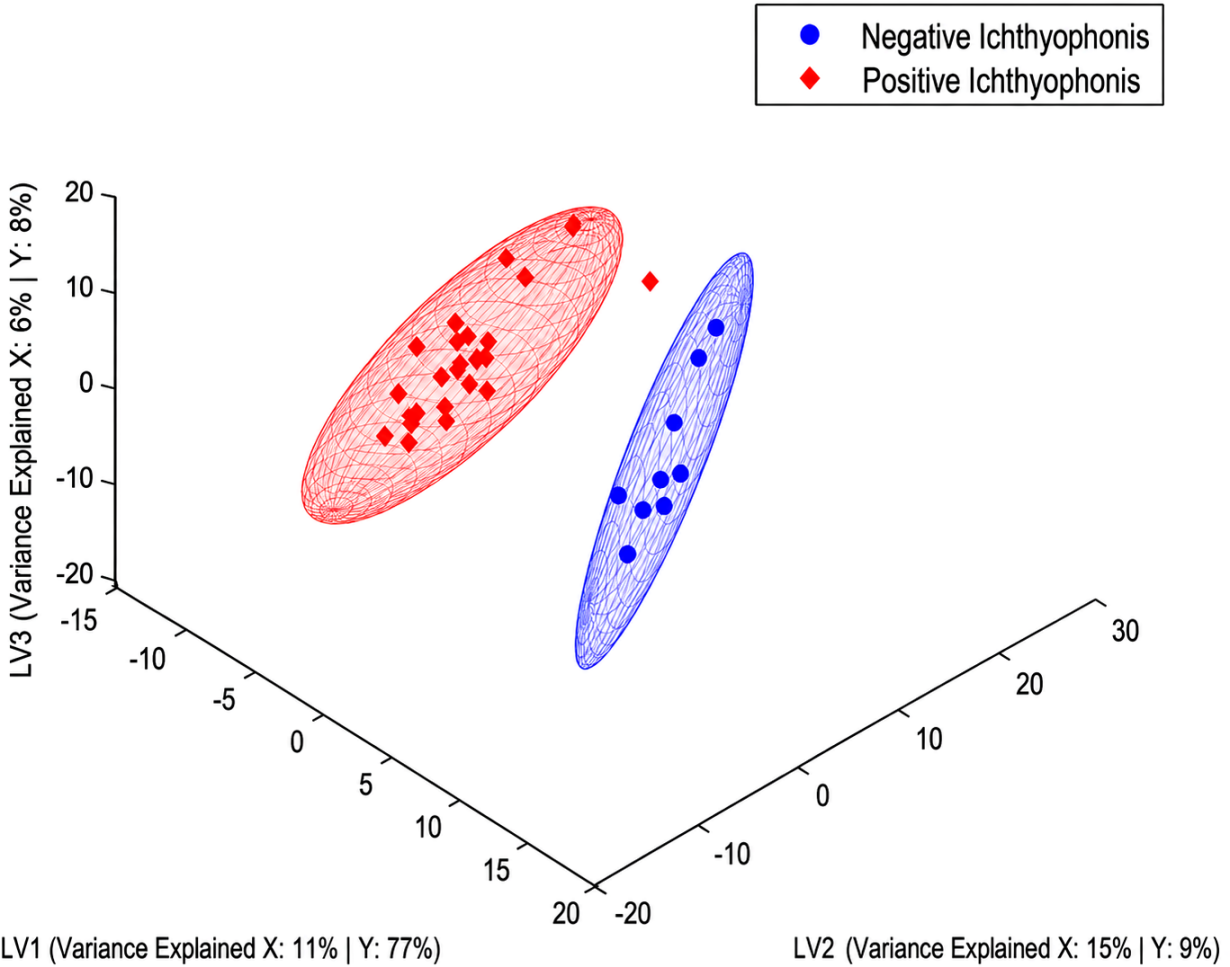
Three dimensional PLS-DA plot shoeing the separation between *I. hoferi-*positive and *I. hoferi-*negative white stumpnose (*Rhabdosargus globiceps)*. The red cluster represents *I. hoferi-*positive fish, while the blue cluster represents uninfected controls. The first three latent variable explained a substantial proportion of variation in the dataset (LV1: X + 11%, Y77%; LV2 X = 15%, Y = 9%; LV3: X = 6%, Y8%), demonstrating clear discrimination between two groups).

The GC×GC-ToF-MS analysis identified 655 metabolites in dried blood spot (DBS) samples collected from *Rhabdosargus globiceps* (White stumpnose). PCA revealed a clear metabolic distinction between the control and *Ichthyophonus hoferi*-infected groups, indicating that infection substantially altered the fish metabolome. The first three principal components (PC1, PC2, and PC3) explained 22%, 10%, and 10% of the variance, respectively (**Figure 1**). PLS-DA enhanced group separation, confirming distinct metabolic profiles between infected and uninfected groups (**Figure 2**). Model performance metrics were as follows: R²X = 32.6%, R²Y = 93.2%, and Q²Y = 59.3%, indicating good predictive capability. Cross-validation confirmed the robustness and reliability of the PLS-DA model.

### Identification of differential metabolites

Differentially abundant metabolites were identified using VIP (Variable Importance in Projection) scores from the PLS-DA model, where VIP ≥ 1.0 was considered significant. From the total dataset, 98 metabolites were identified as contributing substantially to the observed group differences. The 50 most influential metabolites were prioritized as potential biomarkers (**Table 1**). These included several xenobiotics, fatty acids, carbohydrates, and other key analytes.

**Table 1:** Top 50 metabolites potential biomarkers from *I. hoferi-*infected and control White Stumpnose (*Rhabdosargus globiceps)* DBS samples with their respective PLS-DA VIP values, fold changes, chemical classification, and KEGG numbers.

Upregulated metabolites	VIP	Fold change	Chemical class	KEGG number
Cyclohexane	10.94	Up	XEN	KEGG Compound C11249
Propanoic acid	7.47	Up	Carboxylic acid	KEGG Compound C00163
5-Decyne	7.03	Up	XEN	N/A
1,3-Benzenedicarboxylic acid	5.62	Up	FA	N/A
Analyte 482	5.36	Up	N/A	N/A
Pentos-3-ulose	4.48	Up	Carb	N/A
Dotriacontane	3.97	Up	XEN	N/A
Benzoic acid	3.90	Up	XEN	KEGG Compound C00180
Myo-Inositol	3.19	Up	Carb	KEGG Compound C00137
Palmitoleic-Acid	3.16	Up	FA	N/A
Analyte 621	2.884	Up	N/A	N/A
Oxalic acid	2.851	Up	Carb	KEGG Compound C00209
Ribitol	2.691	Up	Carb	KEGG Compound C00474
Nonadecanol	2.677	Up	FA	N/A
D-Pinitol	2.329	Up	FA	KEGG Compound C03844
Heptadecanoic acid	2.173	Up	FA	N/A
9-Tetradecenoic-Acid	2.109	Up	FA	N/A
2-methyloctacosane	1.796	Up	XEN	N/A
Cyclooctasiloxane	1.786	Up	XEN	N/A
Downregulated Metabolites	VIP	Fold Change	Chemical Class	KEGG Number
Phthalic acid	10.95	Down	XEN	KEGG Compound C01606
1,2-Benzenedicarboxylic acid	10.54	Down	XEN	KEGG Compound C01606
Analyte 279	8.86	Down	N/A	N/A
1,2-Benzenedicarboxylic-Acid	7.19	Down	Aromatic dicarboxylic acid	N/A
Arabinofuranose	7.13	Down	Carb	KEGG Compound C06115
Pentadecanoic acid	6.03	Down	FA	KEGG Compound C16537
Hexanedioic acid	5.76	Down	Organic compound	KEGG Compound C06104
Tetraoxa	4.33	Down	XEN	N/A
Adipic-Acid	4.10	Down	FA	N/A
d-Mannose	3.46	Down	Carb	KEGG Compound C00159
Analyte 565	3.27	Down	N/A	N/A
1,1’-Biphenyl, 2,2’,5,5’-tetramethyl	3.06	Down	XEN	N/A
Siloxane	2.722	Down	Xeno	N/A
Oleic acid	2.602	Down	FA	KEGG Compound C00712
Butanedioic acid	2.489	Down	FA	KEGG Compound C00042
Analyte 875	2.473	Down	N/A	N/A
Isopropyl myristate	2.338	Down	XEN	N/A
Methane, di-p-tolyl	2.181	Down	XEN	N/A
Analyte 315	2.173	Down	N/A	N/A
Analyte 823	1.987	Down	N/A	N/A
Benzene	1.852	Down	XEN	KEGG Compound C01407
Pentanedioic acid	1.784	Down	XEN	KEGG Compound C00489
Analyte 940	1.775	Down	XEN	N/A

Among the top altered metabolites, phthalic acid, cyclohexane, propanoic acid, and arabino furanose exhibited notable differences between groups. Several carbohydrates were found to be significantly downregulated in the infected group, including D-mannose, pento-3-ulose, and arabino furanose. In contrast, inositol, ribitol, and oxalic acid were upregulated. Important fatty acids that were altered during infection included pentadecanoic acid, adipic acid, palmitelaidic acid, 9-tetradecenoic acid, heptadecanoic acid, oleic acid, nonadecanol, butanedioic acid, and D-pinitol.

### Pathway enrichment and metabolic impact analysis

**Figure 3:**
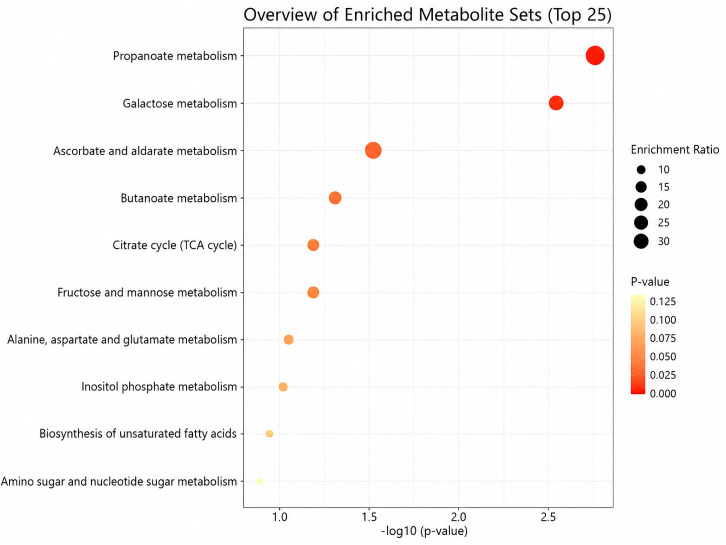
Metabolic pathways associated with the top 50 determined potential biomarkers in white stumpnose fish (*Rhabdosargus globiceps)* infected and uninfected with the *I. hoferi*. The circle size represents the enrichment ratio, and the color represents the p-value.

**Figure 4:**
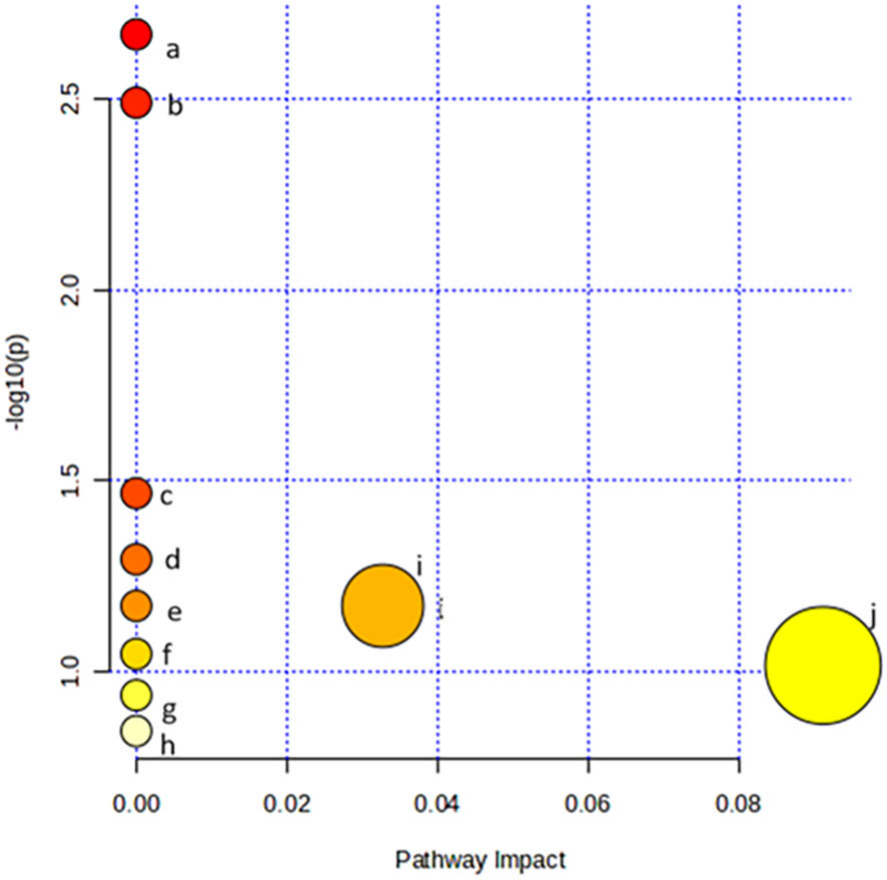
Metabolic pathway analysis (MetPA) illustrating the matched metabolic pathway associated with the top 50 identified metabolic in white stumpnose fish (*Rhabdosargus globiceps*) uninfected and infected with *I. hoferi*. The node size represents the metabolic pathway and is proportional to the enrichment ratio. The color of the nodes represents the level of significance (p-value), from yellow to red (more significant) to yellow (less significant). Key* a- propanoate metabolism; b-galactose metabolism; c-ascorbate and aldarate metabolism; d-Butanoate metabolism; e- fructose and mannose metabolism; f-alanine, aspartate, and glutamate metabolism; g-biosynthesis of unsaturated fatty acids; h-amino sugar and nucleotide sugar metabolism; i- citrate cycle (TCA); and j-inositol phosphate metabolism.

To elucidate the biological relevance of the observed metabolite alterations, pathway analysis was performed using Metabolic Pathway Analysis (MetPA) and Metabolite Set Enrichment Analysis (MSEA). Key pathways significantly perturbed in *I. hoferi*-infected fish included propanoate metabolism, galactose metabolism, and ascorbate and aldarate metabolism (**Figure 3**). In contrast, pathways such as amino acid and nucleotide metabolism, biosynthesis of unsaturated fatty acids, and inositol phosphate metabolism were minimally affected. According to the MetPA metabolome overview (**Figure 4**), the TCA cycle and inositol phosphate metabolism emerged as the most impacted pathways, further highlighting metabolic disruption during infection.

### Identification and verification of potential biomarkers

**Figure 5:**
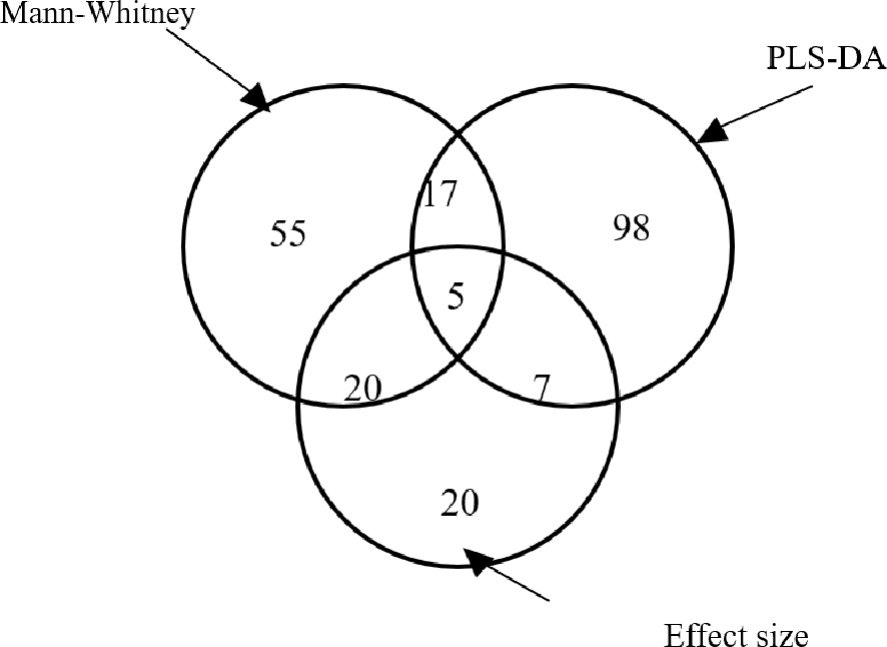
Venn diagram showing metabolites selection using univariate and multivariate statistical tests used to select potential markers of *I. hoferi*-infection in white stumpnose (*Rhabdosargus globiceps*) after *I. hoferi*-infection. Numbers represent the number of metabolites matched for each stat and significant.

To identify robust biomarkers for *I. hoferi* infection, three complementary statistical methods were employed: PLS-DA (VIP ≥ 1.0), Mann-Whitney U test (p ≤ 0.05), and effect size analysis (Cohen’s d ≥ 0.5). A total of 98 metabolites met the PLS-DA threshold, 55 were significant by the Mann-Whitney U test, and 20 showed meaningful effect sizes. Cross-comparison using a Venn diagram (**Figure 5**) revealed five metabolites that met all criteria and were thus identified as strong candidate biomarkers (**Table 2**). All five biomarkers were significantly downregulated in the *I. hoferi*-infected group, with D-mannose exhibiting the most pronounced decrease. These findings suggest potential utility for these metabolites in disease detection and monitoring.

**Table 2:** Metabolite markers that best (metabolite significant in the t-test, PLSDA, Venn diagram and effect size) describe the variation between the control and *I. hoferi-*infected White Stumpnose fish samples.

Compounds	*t-test* p-value	PLS-DA VIP-value	Effect size *d-value*	Positive fish concentration (ng/μL)	Negative fish concentration (ng/μL)
1, 2 Benzenedicarboxylic acid	<0.05	7.970	0.651	1.726 ± 2.414	3.891 ± 1.512
Propanoic acid	<0.05	5.134	0.571	0.304 ± 0.264	0.890 ± 0.319
Analyte 279	<0.05	6.617	0.561	0.636 ± 0.813	0.967 ± 0.237
D-mannose	<0.05	2.187	0.541	15.082 ± 10.033	37.020 ± 20.777
Adipic acid	<0.05	2.597	0.501	0.823 ± 0.513	1.277 ± 0.375

## Discussion

In this study, an untargeted metabolomics approach using comprehensive two-dimensional gas chromatography time-of-flight mass spectrometry (GC×GC-ToF-MS) was employed to characterize metabolic alterations, identify affected biochemical pathways, and determine potential biomarkers associated with *Ichthyophonus hoferi* infection in *Rhabdosargus globiceps* (White stumpnose). Additionally, the chosen platform was ideal for its high sensitivity identifying and quantifying small molecule metabolite, which includes small acids, hydroxyl acids, alcohols, amino acids, toxins, and fatty acids (Fiehn 2016). The results revealed significant differences in metabolite concentrations between infected and uninfected (control) fish, with several key metabolic pathways found to be perturbed during infection, including propanoate metabolism, the tricarboxylic acid (TCA) cycle, inositol phosphate metabolism, and galactose metabolism.

### Propanoate metabolism and inflammatory responses

Among the most enriched pathways, propanoate metabolism was notably upregulated in the *I. hoferi*-infected group. One of its key intermediates, propanoic acid, was significantly elevated in infected fish. Propanoic acid has been implicated in various physiological processes, including protein biosynthesis required for blood coagulation (Luo et al., 2017), modulation of gastrointestinal function (MacFabe et al., 2007), and immune signaling through mast cell activation (Gonzalez-Garcia et al., 2017). Given the role of mast cells in the defense against parasitic infections via histamine release (Lantz et al., 1998; Crivellato and Ribatti, 2010; Beghdadi et al., 2011), the observed upregulation of propanoic acid may indicate a host immune response aimed at parasite containment, possibly contributing to granuloma formation and melanophore accumulation previously described during chronic *Ichthyophonus* infections (Rahimian, 1998).

### TCA cycle and nitrogen metabolism interplay

The TCA cycle, a central metabolic pathway for energy production and cellular respiration, was also affected. Several carbohydrate-related metabolites were downregulated in infected fish, suggesting impaired substrate input into the TCA cycle. Interestingly, urea, a metabolite indirectly linked to the TCA cycle via its connection to the urea cycle, was elevated in infected samples. The intersection of the urea and TCA cycles occurs through fumarate, a shared intermediate. Urea production reflects nitrogen waste management and ammonia detoxification processes particularly important during infection when amino acid catabolism is elevated (Brew et al., 2016). The upregulation of urea may thus suggest increased protein turnover and ammonia detoxification in response to infection stress.

Furthermore, enhanced activity in the D-arginine and D-ornithine metabolism pathway, which contributes to arginine biosynthesis, may indicate a host attempt to maintain nitrogen balance and support immune function. Arginine is known to stimulate anabolic hormone secretion, enhance wound healing, and support immune responses (Barbut and Dawson, 2018), all of which are critical during infection.

### Carbohydrate metabolism and host-parasite interactions

Several carbohydrate metabolites were significantly altered during infection, including D-mannose, pento-3-ulose, and arabinofuranose. D-mannose was notably downregulated in infected fish. As a key energy metabolite and glycoprotein precursor, D-mannose plays a role in immune system activation and structural tissue maintenance (Zhang et al., 2023). Its depletion may reflect parasite-driven consumption, as parasites are known to hijack host red blood cell metabolites for survival, modulating energy production, redox homeostasis, and nucleotide metabolism (Olszewski et al., 2009). Moreover, D-mannose has documented antimicrobial properties and has been shown to prevent hemagglutination in fish (Trust et al., 1981) and alleviate gastrointestinal and metabolic disorders (Altarac and Papeš, 2014). The downregulation of D-mannose could therefore compromise host defense mechanisms against *I. hoferi*, particularly in the gut, where granulomatous lesions are commonly observed (Hershberger et al., 2002).

### Myo-inositol and cell protection mechanisms

Conversely, myo-inositol was significantly upregulated in the infected group. As a precursor of phosphatidylinositol, a critical component of biological membranes, myo-inositol is essential for membrane integrity, signaling, and oxidative responses (Phillips et al., 2006; Schink et al., 2013). Its involvement in inositol phosphate, galactose, and ascorbate and aldarate metabolism underscores its multifaceted role in protecting cells from damage. The elevation of myo-inositol may indicate a compensatory response to cellular damage in infected tissues such as the liver and organs targeted by *I. hoferi*. Additionally, the observed upregulation of D-pinitol, a known precursor of myo-inositol with antioxidant and antimicrobial properties (Haque et al., 2024), supports this protective role.

### Fatty acid dysregulation and inflammation

Alterations in fatty acid profiles further highlight the host’s metabolic response to infection. Oleic acid, a monounsaturated fatty acid with anti-inflammatory properties, was downregulated in infected fish. This reduction could impair the biosynthesis of unsaturated fatty acids, negatively impacting cardiovascular, neuroprotective, and antioxidant functions (Nakamura et al., 2001; Bourre, 2004; Liu et al., 2023). In contrast, D-pinitol and adipic acid showed differential regulation. While D-pinitol was elevated possibly reflecting its antioxidant role adipic acid levels were lower in infected fish. Although adipic acid is slightly toxic to fish (Kennedy, 2002), its role in modulating urinary metabolites in mammals suggests potential impacts on the TCA cycle and cellular metabolism.

### Presence of xenobiotics and environmental exposure

A substantial number of xenobiotic compounds, including phthalic acid, 1,2-benzenedicarboxylic acid, and cyclohexene, were detected in both infected and control fish. Phthalic acid, a common plasticizer in PVC-based materials, was downregulated in the infected group. These compounds are likely of environmental origin, as the samples were collected from aquarium-maintained fish. PVC pipes, cleaning agents, and other materials used in aquarium maintenance can leach phthalates into the water, which are then absorbed by fish through the skin, gills, or ingestion (Xu et al., 2018; Critchell and Hoogenboom, 2018; Przybylińska and Wyszkowski, 2016).

The reduced levels of these xenobiotics in infected fish may reflect differences in exposure, uptake, distribution, or elimination between groups. However, in the absence of direct measurement of detoxification pathways which may include cytochrome P450 enzyme activity or liver histopathology, it is not possible to attribute these differences to specific physiological mechanisms such as altered biotransformation. A study conducted by Coombs, Wolf et al. (1990), reported mice that had *Leishmania donovani* infection had a decreased activities of liver enzymes involved in metabolism of xenobiotics. Additionally, the study reported a decreased cytochrome P450 levels. According to the work conducted by Nambiar et al. (2025), cytochrome P450 enzymes play a crutial role in the biotransformation of both xenobiotics and endogenous compound in fish, thereby connecting environmental exposure to physiological response.

Therefore, these findings should be interpreted cautiously and considered hypothesis-generating. Future studies incorporating targeted assessments of xenobiotics metabolism and tissue pathology would be needed to clarify the underlying processes. Additionally, as no direct measurement of detoxification enzymes activity or liver pathology were conducted in this study, these interpretations remain tentative and need further investigation.

### Interpretative framework, diagnostic potential, and future direction

While the metabolomic alterations identified in this study provide important insight into host–parasite interactions during *Ichthyophonus hoferi* infection in *Rhabdosargus globiceps*, it is important to contextualize these findings within an early-stage biomarker discovery framework. The metabolic features reported here represent candidate infection-associated signatures rather than validated diagnostic biomarkers. Future research should focus on several key directions to advance these findings toward diagnostic application. These include (i) validation of candidate biomarkers in larger and independent fish populations, (ii) comparative metabolomic profiling across multiple pathogens (including phylogenetically related agents such as the rosette agent), (iii) integration with targeted biochemical assays (e.g., enzymatic activity and immune markers), and (iv) exploration of minimally invasive sampling matrices such as blood, mucus, or fin tissue to enable non-lethal diagnostics.

## Conclusion

This study provides the first comprehensive metabolomic profile of *Ichthyophonus hoferi* infection in *Rhabdosargus globiceps* using an untargeted GC×GC-ToF-MS approach. The findings reveal significant alterations in key metabolic pathways, including propanoate metabolism, the TCA cycle, galactose metabolism, and inositol phosphate metabolism, highlighting the systemic impact of *I. hoferi* infection. Several metabolites, including propanoic acid, D-mannose, myo-inositol, oleic acid, and phthalic acid, emerged as potential biomarkers of infection. These results not only contribute to our understanding of host-pathogen metabolic interactions in marine fish but also offer potential biomarkers for early detection, monitoring, and management of ichthyophoniasis in aquaculture and wild populations. However, the findings should be interpreted considering certain limitations. The relatively small sample size may reduce statistical power and generalizability, while the putative nature of metabolite identification (based solely on spectral matching) warrants further structural validation. Moreover, such class imbalance can influence multivariate statistical modelling, particularly supervised approaches such as orthogonal partial least squares discriminant analysis (OPLS-DA), which are sensitive to overfitting. This may lead to exaggerated group separation and reduced reliability of predictive metrics, including Q², which reflects the model’s predictive capacity. Consequently, the observed discrimination between groups should be interpreted with caution. The findings are therefore considered exploratory, and further validation using larger and more balanced sample sizes is required to confirm the robustness and reproducibility of the identified biomarkers.

Additionally, environmental confounding from xenobiotic exposure in the aquarium setting could influence the metabolomic profile, and the cross-sectional design limits understanding of temporal dynamics. Despite these constraints, the study provides a strong foundation for future investigations into diagnostic biomarker development and the molecular mechanisms underlying ichthyophoniasis.

A further limitation of this study is the absence of comparative analysis with other pathogens known to infect the same host species, including phylogenetically related organisms such as the rosette agent. Without such comparisons, it is not possible to determine whether the identified metabolic signatures are specific to *Ichthyophonus hoferi* infection or represent a more general host response to infection or physiological stress.

## Data Availability

The original contributions presented in the study are included in the article/**Supplementary Material**, further inquiries can be directed to the corresponding author/s.
