# Supplementary figures and images for: Identification of metabolic biomarkers and pathways associated with Ichthyophonus hoferi infection in White stumpnose (Rhabdosargus globiceps)
Source: Front Physiol. 2026 Jun 30;17:1826309. doi: 10.3389/fphys.2026.1826309 (PMC13364621; doi:10.3389/fphys.2026.1826309)

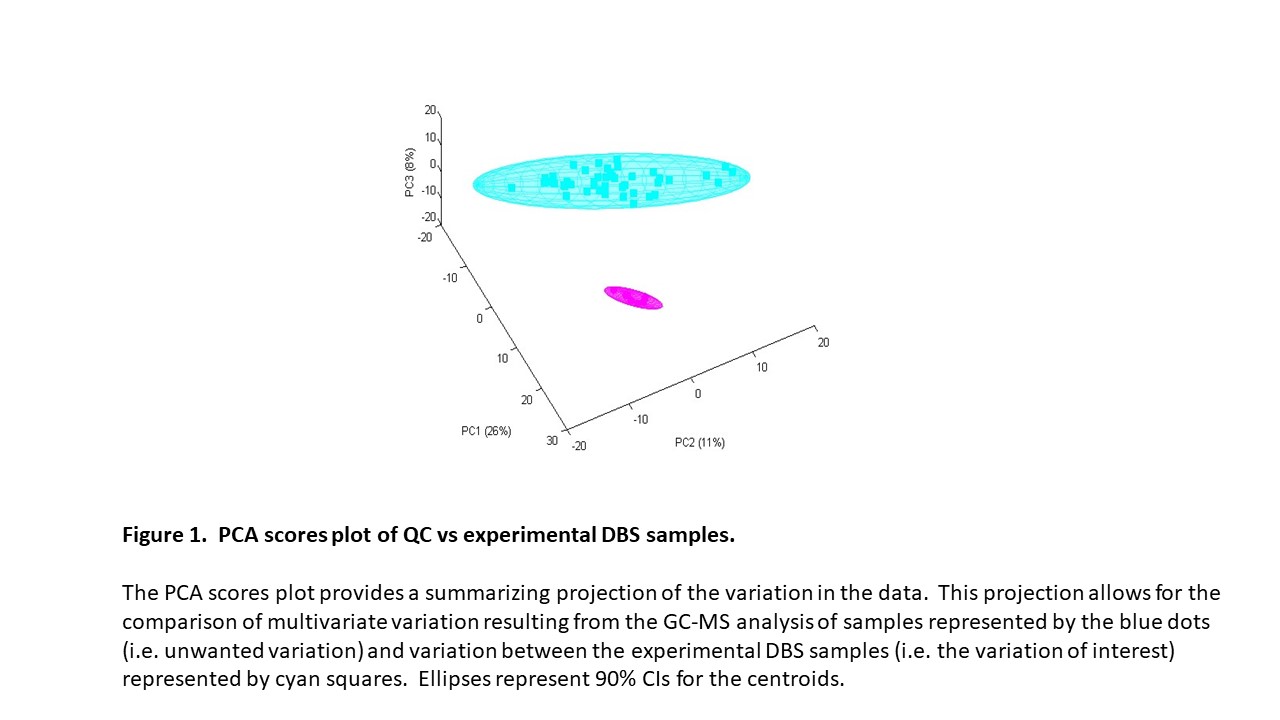

Supplement: Supplementary file 1 [file Image1.jpeg]
