# Supplementary material for: Identification of metabolic biomarkers and pathways associated with Ichthyophonus hoferi infection in White stumpnose (Rhabdosargus globiceps)
Source: Front Physiol. 2026 Jun 30;17:1826309. doi: 10.3389/fphys.2026.1826309 (PMC13364621; doi:10.3389/fphys.2026.1826309)
Supplement: Supplementary file 2 [file DataSheet1.pdf]

Appendix 4

Table 5: The 573 White stump-nose metabolites detected on the DBS incidental sample

(E)-9-Octadecenoic acid ethyl ester(88)	Analyte 138(135)	Analyte 595(221)	D-(-)-Erythrose, tris(trimethylsilyl) ether, methyloxime (anti)(205)
1-(2-Methoxyethoxy)-2-methyl-2-propanol, trimethylsilyl ether(131)	Analyte 14(73)	Analyte 6(89)	D-(-)-Ribofuranose, tetrakis(trimethylsilyl) ether (isomer 1)(217)
1,1,1,5,7,7,7-Heptamethyl-3,3-bis(trimethylsiloxy)tetrasiloxane(73)	Analyte 140(184)	Analyte 605(171)	D-(-)-Ribofuranose, tetrakis(trimethylsilyl) ether (isomer 2)(217)
1,11-Undecanedioic acid, di(trimethylsilyl) ester(345)	Analyte 145(74)	Analyte 61(154)	D-(-)-Tagatose, pentakis(trimethylsilyl) ether, methyloxime (anti)(103)
1,12-Dihydroxydodecane(149)	Analyte 148(98)	Analyte 613(73)	D-(+)-Talofuranose, pentakis(trimethylsilyl) ether (isomer 2)(217)
1,12-Dihydroxyoctadecane(187)	Analyte 149(74)	Analyte 621(293)	D-(+)-Xylose, tetrakis(trimethylsilyl) ether, methyloxime (anti)(73)
1,1'-Biphenyl, 2,2',5,5'-tetramethyl-(195)	Analyte 15(72)	Analyte 625(211)	D-(+)-Xylose, tetrakis(trimethylsilyl) ether, methyloxime (anti)(103)
1,1'-Biphenyl, 3-methyl-(168)	Analyte 157(216)	Analyte 629(73)	D-Arabinonic acid, 2,3,5-tris-O-(trimethylsilyl)-, • -lactone(73)
1,2,3-Propanetricarboxylic acid, 2-[(trimethylsilyl)oxy]-, tris(trimethylsilyl) ester(273)	Analyte 159(179)	Analyte 639(223)	Decanoic acid, trimethylsilyl ester(117)
1,2-Benzenedicarboxylic acid, bis(2-methylpropyl) ester(149)	Analyte 16(248)	Analyte 64(70)	Decanoic-Acid(117)
1,2-Benzenedicarboxylic acid, butyl 2-methylpropyl ester(149)	Analyte 161(74)	Analyte 644(111)	Dehydroabietic acid, trimethylsilyl ester(239)
1,2-Benzenedicarboxylic-Acid(147)	Analyte 162(319)	Analyte 645(117)	D-Erythronic acid • -lactone(147)
1,2-Cyclohexanedicarboxylic acid, cyclohexylmethyl nonyl ester(155)	Analyte 167(158)	Analyte 648(73)	d-Galactose, 2,3,4,5,6-pentakis-O-(trimethylsilyl)-, o-methyloxyme, (1Z)-(73)
1,2-Cyclohexanedicarboxylic acid, dinonyl ester(155)	Analyte 170(74)	Analyte 649(135)	d-Galactose, 2,3,4,5,6-pentakis-O-(trimethylsilyl)-, o-methyloxyme, (1Z)-(217)

1,2-Cyclohexanedicarboxylic acid, heptadecyl 2-methylcyclohexyl ester(155)	Analyte 173(299)	Analyte 65(193)	d-Galactose, 2,3,4,5,6-pentakis-O-(trimethylsilyl)-, o-methyloxyme, (1Z)-(319)
1,2-Dihydroxy-2-Propane(131)	Analyte 176(131)	Analyte 670(132)	D-Glucitol, 6-deoxy-1,2,3,4,5-pentakis-O-(trimethylsilyl)-(117)
1,3-Benzenedicarboxylic acid, bis(2-ethylhexyl) ester(70)	Analyte 179(74)	Analyte 685(143)	D-Glucose, 2,3,4,5,6-pentakis-O-(trimethylsilyl)-(204)
1,3-Benzoxazol-2-amine-ditms(263)	Analyte 18(100)	Analyte 698(213)	d-Glucose, 2,3,4,5,6-pentakis-O-(trimethylsilyl)-, o-methyloxyme, (1Z)-(319)
1,3-Dioxolane(73)	Analyte 181(99)	Analyte 702(159)	D-Glucuronic acid, 2,3,4,5-tetrakis-O-(trimethylsilyl)-, trimethylsilyl ester(217)
1,3-Dioxolane, 2-(1-bromoethyl)-(73)	Analyte 185(74)	Analyte 708(211)	Dibutyl phthalate(149)
1,3-Dioxolane, 2-(3-bromo-5,5,5-trichloro-2,2-dimethylpentyl)-(73)	Analyte 186(59)	Analyte 710(199)	Dicyclopentadiene(66)
1,3-Dioxolane-2-methanol(73)	Analyte 189(73)	Analyte 718(87)	d-Mannose, 2,3,4,5,6-pentakis-O-(trimethylsilyl)-, o-methyloxyme, (1Z)-(160)
1,4-Benzenedicarboxylic acid, bis(trimethylsilyl) ester(295)	Analyte 19(77)	Analyte 72(114)	d-Mannose, 2,3,4,5,6-pentakis-O-(trimethylsilyl)-, o-methyloxyme, (1Z)-(319)
1,4-Butanediamine, N,N,N',N'-tetrakis(trimethylsilyl)-(174)	Analyte 190(133)	Analyte 720(73)	
1,5,9-Undecatriene, 2,6,10-trimethyl-, (Z)-(81)	Analyte 196(240)	Analyte 724(87)	Dodecane, 2,7,10-trimethyl-(71)
1,8-cis-Undecadien-5-yne 3,7-bis-trimethylsilyl ether(129)	Analyte 199(175)	Analyte 726(173)	Dodecanedioic-Acid(55)
1,9-Dihydroxydecane(117)	Analyte 201(59)	Analyte 729(159)	Dodecanoic acid, trimethylsilyl ester(117)
11,14-Eicosadienoic-Acid(67)	Analyte 203(73)	Analyte 730(131)	Dodecyl acrylate(55)
11-Eicosenoic-Acid(129)	Analyte 204(73)	Analyte 732(117)	Dotriacontane(57)
18-Methyl-nonadecanol, trimethylsilyl ether(57)	Analyte 205(73)	Analyte 739(131)	Dotriacontane(71)
1-Docosanol, trimethylsilyl ether(383)	Analyte 206(215)	Analyte 74(57)	D-Pinitol, pentakis(trimethylsilyl) ether(73)
1-Iodo-2-methylundecane(71)	Analyte 214(73)	Analyte 741(103)	D-Pinitol, pentakis(trimethylsilyl) ether(217)

1-Methoxy-5-trimethylsilyloxyhexane(117)	Analyte 216(89)	Analyte 746(87)	Eicosane(57)
1-Monooleoylglycerol trimethylsilyl ether(129)	Analyte 218(73)	Analyte 75(55)	Eicosane(71)
1-O-Hexadecenylglycerol, bis(trimethylsilyl) ether(205)	Analyte 222(73)	Analyte 753(265)	Eicosanoic acid, trimethylsilyl ester(117)
1-O-hexadecylglycerol - bis-trimethylsilyl ether derivative(57)	Analyte 232(89)	Analyte 764(129)	Erythro-Pentonic acid, 2-deoxy-3,4,5-tris-O-(trimethylsilyl)-, trimethylsilyl ester(147)
1-O-hexadecylglycerol - bis-trimethylsilyl ether derivative(205)	Analyte 234(207)	Analyte 766(55)	Ethyl 2,3,4,6-tetrakis-O-(trimethylsilyl)-D-glucopyranoside(204)
1-Propene-1,2,3-tricarboxylic acid, tris(trimethylsilyl) ester, (E)-(229)	Analyte 237(116)	Analyte 769(343)	Ethyl cyclohexanepropionate(88)
1-Trimethylsilyloxytetradecane(271)	Analyte 243(71)	Analyte 771(213)	Ethylenediaminetetraacetic acid, tetrakis(trimethylsilyl) ester(290)
1-Undecene, 11-nitro-(55)	Analyte 249(113)	Analyte 775(129)	Gluconic acid, 2-methoxime, tetra(trimethylsilyl)-, trimethylsilyl ester(204)
2-(2',4',4',6',6',8',8'-Heptamethyltetrasiloxan-2'-yloxy)-2,4,4,6,6,8,8,10,10-nonamethylcyclopentasiloxane(73)	Analyte 258(73)	Analyte 781(73)	Glucopyranose, pentakis-O-trimethylsilyl-(204)
2-(2',4',4',6',6',8',8'-Heptamethyltetrasiloxan-2'-yloxy)-2,4,4,6,6,8,8,10,10-nonamethylcyclopentasiloxane(221)	Analyte 262(207)	Analyte 787(120)	Glycerol, tris(trimethylsilyl) ether(73)
2-(2',4',4',6',6',8',8'-Heptamethyltetrasiloxan-2'-yloxy)-2,4,4,6,6,8,8,10,10-nonamethylcyclopentasiloxane(281)	Analyte 268(71)	Analyte 789(225)	Glycerol, tris(trimethylsilyl) ether(131)
2-(2-Butoxyethoxy)ethoxy-trimethylsilane(116)	Analyte 271(69)	Analyte 79(73)	Glycerol, tris(trimethylsilyl) ether(134)
2,2,11,11-tetramethyl-8-(trimethylsilyloxy)-5-((trimethylsilyloxy)methyl)-3,6,10-trioxa-2,11-disiladodecane(103)	Analyte 274(73)	Analyte 791(117)	Glycerol, tris(trimethylsilyl) ether(147)
2,2,4,7,10-Pentamethyl-3,6,9,12-tetraoxa-2-silatridecane(131)	Analyte 279(145)	Analyte 792(87)	Glycerol, tris(trimethylsilyl) ether(148)
2,2-Bis[(4-trimethylsiloxy)phenyl]propane(357)	Analyte 28(318)	Analyte 794(175)	Glycerol, tris(trimethylsilyl) ether(187)

2,4'-Bipyridine(156)	Analyte 280(71)	Analyte 801(146)	Glycerol, tris(trimethylsilyl) ether(205)
2-[(Trimethylsilyl)oxy]propan-1-ol(117)	Analyte 281(73)	Analyte 803(225)	Glycine, N-(trimethylsilyl)-, trimethylsilyl ester(102)
2-Aminophenol, O-tert.butyldimethylsilyl-(166)	Analyte 282(73)	Analyte 807(73)	Heneicosane(57)
2-Bis(trimethylsilyl)aminoethanesulfonic acid, trimethylsilyl ester(326)	Analyte 284(155)	Analyte 808(130)	Heneicosane(71)
2-Bromotetradecane(71)	Analyte 286(73)	Analyte 811(489)	Heneicosanoic acid, trimethylsilyl ester(117)
2-Butenedioic acid (E)-, bis(2-ethylhexyl) ester(71)	Analyte 289(267)	Analyte 814(73)	Hentriacontane(71)
2-Butenedioic acid (Z)-, bis(trimethylsilyl) ester(73)	Analyte 29(318)	Analyte 816(117)	Heptacosane(57)
2-Butenedioic acid (Z)-, bis(trimethylsilyl) ester(245)	Analyte 291(145)	Analyte 821(73)	Heptacosane(71)
2-Deoxy-galactopyranose, tetrakis(trimethylsilyl)(204)	Analyte 293(155)	Analyte 823(113)	Heptacosane(85)
2-Desoxy-pentos-3-ulose, bis(methoxime),O,O'-bis(trimethylsilyl)-(231)	Analyte 296(73)	Analyte 829(317)	Heptadecane(57)
2-Ethylhexanoic-Acid(73)	Analyte 30(81)	Analyte 836(199)	Heptadecane, 2-methyl-(71)
2-Hydroxyglutaric-Acid(129)	Analyte 308(129)	Analyte 85(192)	Heptadecanoic acid, glycerine-(1)-monoester, bis-O-trimethylsilyl-(385)
2-Hydroxyheptanoic-Acid(173)	Analyte 309(73)	Analyte 850(317)	Heptadecanoic acid, trimethylsilyl ester(117)
2-Hydroxysebacic-Acid(173)	Analyte 311(73)	Analyte 851(215)	Heptadecanoic acid, trimethylsilyl ester(132)
2-Hydroxysebacic-Acid(317)	Analyte 315(447)	Analyte 852(71)	Heptanoic acid, trimethylsilyl ester(187)
2-Ketoisocaproic acid mo-tms(73)	Analyte 316(73)	Analyte 854(159)	Heptasiloxane, hexadecamethyl-(73)
2-Methyl-3-hydroxybutyric-Acid(117)	Analyte 324(73)	Analyte 865(175)	Heptasiloxane, hexadecamethyl-(221)
2-Methylaminomethyl-1,3-dioxolane(73)	Analyte 338(73)	Analyte 868(71)	Hexadecanoic acid, 2,3-bis[(trimethylsilyl)oxy]propyl ester(371)
2-Methylglutaric-Acid(147)	Analyte 346(73)	Analyte 875(146)	Hexadecanoic acid, 3,7,11,15-tetramethyl-, trimethylsilyl ester(159)
2-methyloctacosane(57)	Analyte 347(147)	Analyte 876(217)	Hexadecanoic acid, ethyl ester(88)
2-Mono-9-Octadecenoylglycerol(103)	Analyte 351(240)	Analyte 888(117)	Hexadecanoic acid, trimethylsilyl ester(117)
2-Monopalmitoylglycerol(103)	Analyte 352(235)	Analyte 90(147)	Hexanedioic acid, bis(2-ethylhexyl) ester(129)

2-Monopalmitoylglycerol(129)	Analyte 354(192)	Analyte 905(71)	Hexanoic acid, 5-oxo-, trimethylsilyl ester(145)
2-Monostearylglycerol(129)	Analyte 356(231)	Analyte 909(155)	Hexanoic acid, trimethylsilyl ester(73)
2-O-Glycerol-^-d-galactopyranoside, hexa-TMS(204)	Analyte 364(211)	Analyte 91(73)	Hexanoic acid, trimethylsilyl ester(74)
2-Undecenoic-Acid(117)	Analyte 365(73)	Analyte 938(117)	Hexanoic acid, trimethylsilyl ester(75)
3,26-Dioxa-2,27-disilaoctacosane, 2,2,4,25,27,27-hexamethyl-(117)	Analyte 367(241)	Analyte 939(149)	Hexanoic acid, trimethylsilyl ester(117)
3,4-Dimethyl-1-pentamethyldisilyloxycyclohexane (147)	Analyte 372(73)	Analyte 940(57)	Hexanoic acid, trimethylsilyl ester(129)
3,6,9,12-Tetraoxa-2,13-disilatetradecane, 2,2,13,13-tetramethyl-(117)	Analyte 38(82)	Analyte 948(57)	Hexanoic acid, trimethylsilyl ester(173)
3,6,9-Trioxa-2,10-disilaundecane, 2,2,10,10-tetramethyl-(73)	Analyte 380(69)	Analyte 954(243)	Hexasiloxane, tetradecamethyl-(221)
3,6,9-Trioxa-2,10-disilaundecane, 2,2,10,10-tetramethyl-(117)	Analyte 388(171)	Analyte 955(243)	Hexasiloxane, tetradecamethyl-(355)
3,9-Dioxa-2,10-disilaundecane, 2,2,10,10-tetramethyl-5-[(trimethylsilyl)oxy]-(143)	Analyte 39(82)	Analyte 956(357)	Hydantoin, 5-hydroxy-tris-O-(trimethylsilyl)-(147)
3-Amino-2-hydroxy-3-phenylbutyramide(120)	Analyte 391(73)	Analyte 958(427)	Hydroxymalonic-Acid(147)
3-Hydroxydodecanedioic-Acid-triTMS(233)	Analyte 393(73)	Analyte 961(129)	Inosine, tetrakis(trimethylsilyl) ether(217)
3-Hydroxydodecanoic-Acid(triTMS)(71)	Analyte 402(221)	Analyte 99(207)	Inositol, 1,2,3,4,5,6-hexakis-O-(trimethylsilyl)-, scyllo-(217)
3-Isopropoxy-1,1,1,7,7,7-hexamethyl-3,5,5-tris(trimethylsiloxy)tetrasiloxane(73)	Analyte 412(73)	Arabinofuranose, 1,2,3,5-tetrakis-O-(trimethylsilyl)-(217)	Isopropyl myristate(60)
3-Isopropoxy-1,1,1,7,7,7-hexamethyl-3,5,5-tris(trimethylsiloxy)tetrasiloxane(221)	Analyte 413(521)	Arachidonic-Acid(91)	L-(-)-Sorbofuranose, pentakis(trimethylsilyl) ether(217)
3-Isopropoxy-1,1,1,7,7,7-hexamethyl-3,5,5-tris(trimethylsiloxy)tetrasiloxane(281)	Analyte 414(73)	Aucubin, hexakis(trimethylsilyl) ether(361)	L-(-)-Sorbose, pentakis(trimethylsilyl) ether, methyloxime (syn)(103)
3-Ketosebacic-Acid(73)	Analyte 417(172)	Azelaic acid, bis(trimethylsilyl) ester(55)	LACTIC-ACID(58)
3-Nonanol, trimethylsilyl eher(131)	Analyte 418(73)	Benzene, 1,2,3-tris[(trimethylsilyl)oxy]-(239)	LACTIC-ACID(134)

3-Octenoic acid, trimethylsilyl ester(199)	Analyte 42(83)	Benzene, 1,2-dimethyl-4-(phenylmethyl)-(181)	l-Alanine, N-(trimethylsilyl)-, trimethylsilyl ester(116)
4,4'-Dimethylbiphenyl(182)	Analyte 424(262)	Benzene, 1-methyl-4-(phenylmethyl)-(167)	L-Aspartic acid, N-(trimethylsilyl)-, bis(trimethylsilyl) ester(232)
4,4'-oxybis(methylene)bis(2,2,7,7-tetramethyl-3,6-dioxa-2,7-disilaoctane)(103)	Analyte 426(73)	Benzoic acid trimethylsilyl ester(147)	L-Isoleucine, N-(trimethylsilyl)-, trimethylsilyl ester(158)
4,7-Bis(trimethylsiloxy)-2,4,7,9-tetramethyl-5-decyne(313)	Analyte 432(221)	Benzoic acid trimethylsilyl ester(179)	L-Leucine, N-(trimethylsilyl)-, trimethylsilyl ester(158)
4,8,12-Trimethyltridecan-4-olide(99)	Analyte 435(117)	Benzoic acid, 2-[(trimethylsilyl)amino]-, trimethylsilyl ester(266)	L-Norleucine, N-(trimethylsilyl)-, trimethylsilyl ester(158)
5-(Hydroxymethyl)Furan-2-Carboxylic-Acid(271)	Analyte 436(274)	Benzoic acid, 4-[(trimethylsilyl)oxy]-, trimethylsilyl ester(223)	l-Norvaline, n-propargyloxycarbonyl-, propargyl ester(154)
5-Hydroxyhexanoic-Acid(117)	Analyte 438(73)	Bis(2-ethylhexyl) phthalate(149)	L-Proline, 1-(trimethylsilyl)-, trimethylsilyl ester(142)
7-Hydroxyoctanoic-Acid(55)	Analyte 448(73)	Bis(2-furfuryl)disulfide(81)	L-Proline, 1-(trimethylsilyl)-4-[(trimethylsilyl)oxy]-, trimethylsilyl ester, trans-(230)
9,12,15-Octadecatrienoic acid, methyl ester, (Z,Z,Z)-(67)	Analyte 449(103)	Bis(trimethylsilyl) 3,6,9,12,15,18,21-heptaoxatricosane-1,23-dioate(175)	L-Proline, 5-oxo-1-(trimethylsilyl)-, trimethylsilyl ester(156)
9,12-Octadecadienoic acid (Z,Z)-, trimethylsilyl ester(67)	Analyte 45(117)	Bis[di(trimethylsiloxy)phenylsiloxy]trimethylsiloxyphenylsiloxane(135)	L-Valine, N-(trimethylsilyl)-, trimethylsilyl ester(144)
9,12-Octadecadienoic acid (Z,Z)-, trimethylsilyl ester(127)	Analyte 452(117)	Bis-1,2-propanediol phosphate, tris(trimethylsilyl)-deriv.(73)	Malic-Acid(73)
9,12-Octadecadienoic acid, methyl ester, (E,E)-(67)	Analyte 454(211)	Butanal, 2,3,4-tris[(trimethylsilyl)oxy]-, O-methyloxime, [R-(R*,R*)]-(205)	Mannonic-Acid(217)
9-Octadecenamide, (Z)-(59)	Analyte 455(73)	Butane, 1,2,3-tris(trimethylsiloxy)-(117)	Mercaptoacetic acid, bis(trimethylsilyl)-(73)
9-Octadecenoic acid, 2-[(trimethylsilyl)oxy]-1-	Analyte 459(73)	Butanedioic acid, bis(trimethylsilyl) ester(147)	Mercaptoacetic acid, bis(trimethylsilyl)-(221)

[[(trimethylsilyl)oxy]methyl]ethyl ester(97)			
9-Tetradecenoic-Acid(117)	Analyte 465(73)	Butanoic acid, 2-[(trimethylsilyl)oxy]-, trimethylsilyl ester(131)	meso-Erythritol, tetrakis(trimethylsilyl) ether(217)
‡-Alanine, N,N-bis(trimethylsilyl)-, trimethylsilyl ester(174)	Analyte 466(217)	Butanoic acid, 3-[(trimethylsilyl)oxy]-, trimethylsilyl ester(147)	Methane, di-p-tolyl-(181)
Acetic acid, [(trimethylsilyl)oxy]-, trimethylsilyl ester(58)	Analyte 47(73)	Butanoic acid, 4-[(trimethylsilyl)oxy]-, trimethylsilyl ester(147)	Methoxydi(tert-butyl)silane(89)
Acetic acid, phenoxy-, trimethylsilyl ester(73)	Analyte 482(117)	Butanoic acid, 4-[bis(trimethylsilyl)amino]-, trimethylsilyl ester(174)	Methyl dehydroabietate(239)
‡-D-(+)-Talopyranose, pentakis(trimethylsilyl) ether(204)	Analyte 483(69)	Cadaverine, N,N,N',N'-tetrakis(trimethylsilyl)(174)	Methyl stearate(87)
‡-D-Glucopyranose, 1,2,3,4,6-pentakis-O-(trimethylsilyl)-(204)	Analyte 485(312)	Carbonic acid, 2-dimethylaminoethyl propyl ester(58)	Methylephedrine(72)
Adipic-Acid(diTMS)(111)	Analyte 489(73)	Cholest-5-en-3-ol (3‡)-, carbonochloridate(105)	Monooleoylglycerol(55)
Alanine, phenyl-, trimethylsilyl ester, dl-(120)	Analyte 495(595)	Cholesta-4,6-dien-3-ol, (3‡)-(135)	Monooleoylglycerol(129)
‡-l-Galactopyranoside, methyl 6-deoxy-2,3,4-tris-O-(trimethylsilyl)-(204)	Analyte 496(73)	cis-13-Docosenoic acid, trimethylsilyl ester(117)	Monopentadecanoylglycerol(357)
Allonic acid, 2,3,5,6-tetrakis-O-(trimethylsilyl)-, lactone(204)	Analyte 500(73)	cis-13-Docosenoic acid, trimethylsilyl ester(129)	Monostearylglycerol(57)
Allonic acid, 2,3,5,6-tetrakis-O-(trimethylsilyl)-, lactone(361)	Analyte 506(96)	cis-15-Tetracosenoic acid, trimethylsilyl ester(117)	Myo-Inositol, 1,2,3,4,5,6-hexakis-O-(trimethylsilyl)-(217)
ˆ-Methyl-L-tyrosine trisItrimethylsilyl)(232)	Analyte 512(160)	cis-4,7,10,13,16,19-Docosahexaenoic acid, trimethylsilyl ester(91)	Myo-Inositol, 1,2,3,4,5,6-hexakis-O-(trimethylsilyl)-(231)
Amine, N,N,N-tris((trimethylsilyloxy)ethyl)-(262)	Analyte 513(105)	cis-5,8,11,14,17-Eicosapentaenoic acid, trimethylsilyl ester(91)	Myristic acid, 2,3-bis(trimethylsiloxy)propyl ester(343)
Aminomalonic acid, tris(trimethylsilyl)-(218)	Analyte 514(73)	cis-5,8,11,14,17-Eicosapentaenoic acid, trimethylsilyl ester(129)	N,N-bis [2-Trimethylsiloxyethyl] ethanamine(174)

Analyte 10(61)	Analyte 515(105)	cis-9-Hexadecenoic acid, trimethylsilyl ester(117)	N,N-Dimethyl-2-isopropoxyethylamine(58)
Analyte 101(207)	Analyte 52(225)	Creatinine enol N1,N3,O-tris(trimethylsilyl)(115)	N,O,O-Tris(trimethylsilyl)-L-threonine(219)
Analyte 103(73)	Analyte 525(105)	Cyclohexasiloxane, dodecamethyl-(73)	N,O-Bis(trimethylsilyl)-L-phenylalanine(218)
Analyte 104(129)	Analyte 527(345)	Cyclohexasiloxane, dodecamethyl-(341)	Naphthalene, 1,2,3-trimethyl-4-propenyl-, (E)-(195)
Analyte 105(74)	Analyte 529(173)	Cyclononasiloxane, octadecamethyl-(73)	Naphthalene, 1,4,5-trimethyl-(155)
Analyte 108(132)	Analyte 53(225)	Cyclononasiloxane, octadecamethyl-(355)	Naphthalene, 1,6,7-trimethyl-(155)
Analyte 110(132)	Analyte 533(131)	Cyclooctasiloxane, hexadecamethyl-(355)	Naphthalene, 1,7-dimethyl-(141)
Analyte 112(146)	Analyte 537(288)	Cyclopentasiloxane, decamethyl-(267)	Naphthalene, 2,3,6-trimethyl-(155)
Analyte 113(74)	Analyte 538(244)	Cyclopentasiloxane, decamethyl-(355)	Naphthalene, 2,6-dimethyl-(141)
Analyte 114(73)	Analyte 54(147)	Cyclopentasiloxane, decamethyl-(356)	Nonadecane(57)
Analyte 115(73)	Analyte 540(117)	Cyclotetrasiloxane, octamethyl-(281)	Nonadecane, 2-methyl-(71)
Analyte 116(207)	Analyte 543(157)	D-(-)-Erythrose, tris(trimethylsilyl) ether, methyloxime (anti)(143)	Nonadecanoic acid, trimethylsilyl ester(117)
Analyte 12(75)	Analyte 544(157)	Analyte 572(318)	Nonanoic acid, trimethylsilyl ester(117)
Analyte 124(73)	Analyte 548(145)	Analyte 573(83)	Nonanoic-Acid(73)
Analyte 125(73)	Analyte 55(147)	Analyte 58(163)	n-Pentadecanoic acid, trimethylsilyl ester(117)
Analyte 126(221)	Analyte 551(157)	Analyte 584(187)	n-Pentadecanoic acid, trimethylsilyl ester(132)
Analyte 13(89)	Analyte 557(236)	Analyte 586(205)	n-Pentadecanoic acid, trimethylsilyl ester(299)
Analyte 135(74)	Analyte 56(163)	Analyte 590(187)	n-Tridecanoic acid, trimethylsilyl ester(117)
Analyte 136(100)	Analyte 565(71)	Octanedioic acid, bis(trimethylsilyl) ester(73)	n-Tridecanoic acid, trimethylsilyl ester(132)
Octacosane(57)	Octanoic acid, trimethylsilyl ester(69)	Octanoic acid, trimethylsilyl ester(132)	Octanoic acid, trimethylsilyl ester(174)
